# Supplementary material for: Special care dentistry perception among dentists in Jakarta: An online survey study
Source: PLoS One. 2021 Apr 7;16(4):e0249727. doi: 10.1371/journal.pone.0249727 (PMC8026065; doi:10.1371/journal.pone.0249727)

PLOS ONE

Special Care Dentistry Perception among Dentists in Jakarta: An Online Survey Study

--Manuscript Draft--

**Manuscript Number:**	PONE-D-20-32506
**Article Type:**	Research Article
**Full Title:**	Special Care Dentistry Perception among Dentists in Jakarta: An Online Survey Study
**Short Title:**	Special Care Dentistry Perception among Dentists in Jakarta
**Corresponding Author:**	Masita Mandasari, D.D.S., Ph.D, Oral Medicine Specialist<br>Universitas Indonesia<br>Jakarta Pusat, Jakarta INDONESIA
**Keywords:**	Dental care for patients with disability; Dental education; Special Care Dentistry; Special Needs Dentistry
**Abstract:**	Special Care Dentistry (SCD) or Special Needs Dentistry is a branch of dentistry concerned with the oral health of people with a variety of medical conditions or limitations that require more than routine delivery of care. There were reports on oral status of special care patients and special interest group for SCD dentists in Indonesia has existed. However, there was not perception report on SCD amongst dentists in Jakarta. This paper will describe the perception of dentists in Jakarta towards SCD. A cross-sectional questionnaire was distributed online through Whatsapp to dentists registered in Jakarta. The questionnaire explored dentists' perception towards SCD. A total of 250 dentists participated in this study, of them 173 general practitioners and 77 specialist dentists. Most respondents reported that they did not have SCD component during undergraduate dental school and did not provide treatment to patients with special needs in their clinical practice. Most respondents have poor perception of SCD, however, most of the respondents showed motivation and interest towards SCD training. Dentists in Jakarta involved in this study had poor perception of SCD. More efforts should be performed to improve SCD education and awareness.
**Order of Authors:**	Masita Mandasari, D.D.S., Ph.D, Oral Medicine Specialist
	Febrina Rahmayanti
	Hajer Derbi
	Yuniardini Septorini Wimardhani
**Additional Information:**	
**Question**	**Response**
**Financial Disclosure**<br><br>Enter a financial disclosure statement that describes the sources of funding for the work included in this submission. Review the submission guidelines for detailed requirements. View published research articles from *PLOS ONE* for specific examples.<br><br>This statement is required for submission and **will appear in the published article** if the submission is accepted. Please make sure it is accurate.	MM received fundings from Universitas Indonesia through International-Indexed Publication Grant (PUTI Universitas Indonesia NKB-1921/UN2.RST/HKP.05.00/2020) https://ui.ac.id/en/. The funders had no role in study design, data collection and analysis, decision to publish, or preparation of the manuscript.

**Unfunded studies**<br>Enter: *The author(s) received no specific funding for this work.*<br><br>**Funded studies**<br>Enter a statement with the following details:<br>• Initials of the authors who received each award<br>• Grant numbers awarded to each author<br>• The full name of each funder<br>• URL of each funder website<br>• Did the sponsors or funders play any role in the study design, data collection and analysis, decision to publish, or preparation of the manuscript?<br>• **NO** - Include this sentence at the end of your statement: *The funders had no role in study design, data collection and analysis, decision to publish, or preparation of the manuscript.*<br>• **YES** - Specify the role(s) played.<br><br>* typeset	
**Competing Interests**<br><br>Use the instructions below to enter a competing interest statement for this submission. On behalf of all authors, disclose any competing interests that could be perceived to bias this work—acknowledging all financial support and any other relevant financial or non-financial competing interests.<br><br>This statement **will appear in the published article** if the submission is accepted. Please make sure it is accurate. View published research articles from *PLOS ONE* for specific examples.	The authors have declared that no competing interests exist.

Powered by Editorial Manager® and ProduXion Manager® from Aries Systems Corporation

**NO authors have competing interests**<br><br>Enter: *The authors have declared that no competing interests exist.*<br><br>**Authors with competing interests**<br><br>Enter competing interest details beginning with this statement:<br><br>*I have read the journal's policy and the authors of this manuscript have the following competing interests: [insert competing interests here]*<br><br>* typeset	
**Ethics Statement**<br><br>Enter an ethics statement for this submission. This statement is required if the study involved:<br><br>• Human participants<br>• Human specimens or tissue<br>• Vertebrate animals or cephalopods<br>• Vertebrate embryos or tissues<br>• Field research<br><br>Write "N/A" if the submission does not require an ethics statement.<br><br>General guidance is provided below. Consult the submission guidelines for detailed instructions. **Make sure that all information entered here is included in the Methods section of the manuscript.**	This study was reviewed and approved by the Ethical Committee Faculty of Dentistry Universitas Indonesia No. 106/Ethical Approval/FKGUI/IX/2019.

Powered by Editorial Manager® and ProduXion Manager® from Aries Systems Corporation

**Format for specific study types**<br><br>**Human Subject Research (involving human participants and/or tissue)**<br>• Give the name of the institutional review board or ethics committee that approved the study<br>• Include the approval number and/or a statement indicating approval of this research<br>• Indicate the form of consent obtained (written/oral) or the reason that consent was not obtained (e.g. the data were analyzed anonymously)<br><br>**Animal Research (involving vertebrate animals, embryos or tissues)**<br>• Provide the name of the Institutional Animal Care and Use Committee (IACUC) or other relevant ethics board that reviewed the study protocol, and indicate whether they approved this research or granted a formal waiver of ethical approval<br>• Include an approval number if one was obtained<br>• If the study involved *non-human primates*, add *additional details* about animal welfare and steps taken to ameliorate suffering<br>• If anesthesia, euthanasia, or any kind of animal sacrifice is part of the study, include briefly which substances and/or methods were applied<br><br>**Field Research**<br><br>Include the following details if this study involves the collection of plant, animal, or other materials from a natural setting:<br>• Field permit number<br>• Name of the institution or relevant body that granted permission	
**Data Availability**<br><br>Authors are required to make all data underlying the findings described fully available, without restriction, and from the time of publication. PLOS allows rare exceptions to address legal and ethical concerns. See the PLOS Data Policy and FAQ for detailed information.	No - some restrictions will apply

A Data Availability Statement describing where the data can be found is required at submission. Your answers to this question constitute the Data Availability Statement and **will be published in the article**, if accepted.<br><br>**Important:** Stating 'data available on request from the author' is not sufficient. If your data are only available upon request, select 'No' for the first question and explain your exceptional situation in the text box.<br><br>Do the authors confirm that all data underlying the findings described in their manuscript are fully available without restriction?	
**Describe where the data may be found in full sentences. If you are copying our sample text, replace any instances of XXX with the appropriate details.**<br><br>• If the data are **held or will be held in a public repository**, include URLs, accession numbers or DOIs. If this information will only be available after acceptance, indicate this by ticking the box below. For example: *All XXX files are available from the XXX database (accession number(s) XXX, XXX.)*.<br>• If the data are all contained **within the manuscript and/or Supporting Information files**, enter the following: *All relevant data are within the manuscript and its Supporting Information files.*<br>• If neither of these applies but you are able to provide **details of access elsewhere**, with or without limitations, please do so. For example:<br><br>*Data cannot be shared publicly because of [XXX]. Data are available from the XXX Institutional Data Access / Ethics Committee (contact via XXX) for researchers who meet the criteria for access to confidential data.*<br><br>*The data underlying the results presented in the study are available from (include the name of the third party*	Data cannot be shared publicly because of the confidentiality clause related to respondent's personal information. Data are available from the Universitas Indonesia Institutional Data Access / Ethics Committee (contact via masitamandasari@ui.ac.id) for researchers who meet the criteria for access to confidential data.

Powered by Editorial Manager® and ProduXion Manager® from Aries Systems Corporation

and contact information or URL).<br>• This text is appropriate if the data are owned by a third party and authors do not have permission to share the data.<br><br>* typeset	
Additional data availability information:	Tick here if the URLs/accession numbers/DOIs will be available only after acceptance of the manuscript for publication so that we can ensure their inclusion before publication.

Manuscript

Click here to access/download;Manuscript;Manuscript SCD - PLoS One.docx

Special care dentistry perception among dentists in Jakarta: An online survey study

Masita Mandasari[1,2*], Febrina Rahmayanti[2], Hajer Derbi[3], Yuniardini S. Wimardhani[2]

[1] Oral Medicine Residency Program, Faculty of Dentistry Universitas Indonesia, Jakarta, Indonesia

[2] Oral Medicine Department, Faculty of Dentistry Universitas Indonesia, Jakarta, Indonesia

[3] Convener, DCD Special Needs Dentistry Program, Melbourne Dental School, The University of Melbourne, Victoria, Australia

* Corresponding author

Email: masitamandasari@ui.ac.id

Abstract

Special Care Dentistry (SCD) or Special Needs Dentistry is a branch of dentistry concerned with the oral health of people with a variety of medical conditions or limitations that require more than routine delivery of care. There were reports on oral status of special care patients and special interest group for SCD dentists in Indonesia has existed. However, there was not perception report on SCD amongst dentists in Jakarta. This paper will describe the perception of dentists in Jakarta towards SCD. A cross-sectional questionnaire was distributed online through Whatsapp to dentists registered in Jakarta. The questionnaire explored dentists' perception towards SCD. A total of 250 dentists participated in this study, of them 173 general practitioners and 77 specialist dentists. Most respondents reported that they did not have SCD component during undergraduate dental school and did not provide treatment to patients with special needs in their clinical practice. Most respondents have poor perception of SCD, however, most of the respondents showed motivation and interest towards SCD training. Dentists in Jakarta involved in this study had poor perception of SCD. More efforts should be performed to improve SCD education and awareness.

Introduction

Oral health was reported to be a significant health problem in Indonesian population.[1] Special Care Patients (SCPs) are in particular more vulnerable to oral health problems than those without, being reported to have higher plaque scores and caries prevalence, as well as poor periodontal health.[2–7] Maintaining good oral hygiene and access to dental care amongst this cohort was, and still is, often non-existent, especially when compared to the general population. Problems such as lack of oral health awareness amongst caregivers and/or patients,

limitation in the ability to perform oral hygiene practices, agility, and poor motor coordination, diet problems, medications' side effects, limited access to dental care, or needing assistance in performing oral hygiene regime are evident to be responsible for the poor oral health in people with disability.[8–10] According to the Ministry of Health Indonesia 2014 report, it is estimated that 2.45% of Indonesian population are living with disability.[11] With a total population of Indonesia around 270 million in 2019[12] accompanied by the current advanced medical care, improved standard of living, healthy lifestyle initiatives and increased life expectancy, there are concerns about the capacity to provide adequate dental services for the elderly as their numbers are increasing and that they are retaining more teeth.[13]

Special Care Dentistry (SCD) or Special Needs Dentistry (SND) is a branch of dentistry concerned with the oral health of people with a variety of medical conditions or limitations that require more than routine delivery of care.[14] The terms 'Special Needs Dentistry' and 'Special Care Dentistry' are used interchangeably on a global level.[13] Not only that there is discrepancy in its nomenclature, but also variation in definition among different countries and even among professional dental societies. Australia and New Zealand use "Special Needs Dentistry" while Europe, the UK, and the US use "Special Care Dentistry".[15,16] In a broader term, SCD focuses on the clinicians' caring for the patients, whereas SND focuses on the patients' needs rather than the care given. The Special Care Dentistry Association (SCDA) defined SCD as "branch of dentistry which provides oral care services for people with physical, medical, developmental, or cognitive conditions, which limit their ability to receive routine dental care".[17] The term SCD will be used onward in this paper.

Special Care Dentistry has been officially recognized as a specialty in Brazil, United Kingdom, Malaysia, New Zealand, and Australia.[15,18–21] In Australia and Malaysia, the number of dentists specializing in SCD are scarce thus it can be assumed that SCPs are often treated by general practitioners.[13,22] It is evident that despite the number of training programs in SCD

becoming available around the world, there will still be some time before there are sufficient numbers to cater to patients demand. Therefore, it is imperative to improve dental trainings so graduates can meet some of the demand.[23–26]

Jakarta is the capital city of Indonesia and according to the Indonesian Dentist Association, has the highest number of dentists in Indonesia, including general practitioners and dentists in various specialties. Special care dentistry has not been recognized as a specialty in Indonesia yet, however, the Indonesian Dentist Association has acknowledged the Indonesian Society of Special Care Dentistry (ISSCD) which is a special interest group gathering dentists who have interest in SCD[27] and currently has a relatively small number of members. It can be inferred that in Indonesia, this patient cohort would predominately be managed by the general dentist, making a study into aspects of their perception with this patient group pertinent. This paper will describe the perception of dentists in Jakarta towards SCD.

Methods

The present study employed a previously validated survey format.[13] This cross-sectional study was targeting dentists who were registered members of Indonesian Dental Association in five cities of Jakarta Special Capital Region in their respective Whatsapp chat groups (East Jakarta, South Jakarta, West Jakarta, North Jakarta, and Central Jakarta). The administrator of each group chat helped to deliver the questionnaire invitation and the reminders to participate on the third and fifth day after the initial invitation. Dentists registered as members of Indonesian Society of Special Care Dentistry, received dental qualification from foreign dental institutions, and those not practicing dentistry were excluded from this study. Respondent’s participation was voluntary, self-recruit, and confidential.

The questionnaire was cross-adapted to Indonesian language according to the method published by Beaton, et al.[28] from the original questionnaire[13] which was in English. The

questionnaire contained questions with yes-or-no and open-ended answers, and Likert Scale items. There were six aspects consisting of demographic characteristics, perception of SCD, perceptions of special needs patients, government programs and initiatives for dentistry in SCD, training programs for SCD, and referral of SCPs to specialist dentists. The questionnaire was modified to include additional items regarding awareness of ISSCD. Based on our consultation with ISSCD, neither "Special Care Dentistry" nor "Special Needs Dentistry" were translated into Indonesian since there has not been a consensus for translation/equivalent terminology.

Before the conduction of the main study, the questionnaire was tested for validity and reliability analysis in small group of respondents (N=33). Validity was tested using face to face validity and reported good comprehension among respondents. Internal reliability was assessed using Cronbach's alpha on questionnaire items using Likert scale. The Cronbach's alpha scores for each item was >0.7 so it can be interpreted that the item was reliable. Retest reliability was assessed using intraclass correlation coefficient obtaining score of 0.818 indicating good reliability.

Statistical analysis of quantitative data was performed using IBM SPSS software (IBM Corp, Armonk, New York, USA). Univariate analysis described the proportion of each variables and bivariate analysis described correlations between variables using Chi-Square test. This study was reviewed and approved by the Ethical Committee Faculty of Dentistry Universitas Indonesia and acknowledged by the Indonesian Dental Society in DKI Jakarta and ISSCD.

Results

Demographic characteristics

The survey was delivered to 1231 dentists in Jakarta. A total of 250 respondents completed the survey which made the response rate 20.3%. As many as 173 (69.2%) respondents were general practitioner dentists and 77 (30.8%) were specialist dentists. The majority of respondents were aged between 24-71 years with the median age of 33. Most respondents were female (83.2%) and had been practicing dentistry for 5-10 years (40.8%). Among specialist dentists, there were 16 (20.8%) orthodontists, 5 (6.5%) oral medicine specialists, 15 (19.5%) pediatric dentistry specialists, 19 (24.7%) endodontist/conservative dentistry specialists, 3 (3.9%) periodontists, 16 (20.8) prosthodontists, and 3 (3.9%) oral and maxillofacial surgeon.

Perception of special care dentistry

The majority of respondents (N=163, 65.2%) reported that they did not receive SCD component during their undergraduate study. Among those respondents who reported that they had SCD component (N=87, 34.8%), the majority (N=70, 80.5%) rated themselves as competent in SCD, ranging from “somewhat competent” to “completely competent”. There was no significant association between the university at which undergraduate training was obtained nor the year of graduation with regards to the SND training.

Over half of the respondents (N=140, 56%) claimed that they were able to define SCD. Of those respondents who said yes, there were asked to provide the definition of SCD. If the respondent mentioned any group of patients who might need SCD (e.g., patients with physical disability, patients with intellectual disability, patients with medical condition, psychiatric issues) the respondent was classified as having 'adequate' knowledge in the SCD field and good

SCD perception. Respondents who only translated SCD or SND into Indonesian language or did not provide any answer were classified into groups of poor SCD perception. Figure 1 shows the frequency of SCD patient groups mentioned by respondents. Only 104 (41.6%) respondents were able to define SCD, patients with psychiatric issues was mentioned the most (N=49) by respondents while patients with sensory impairments was only mentioned once. Medically compromised patients were mentioned by 21 respondents.

Figure 1. Frequency of SCD Patients. Respondent who wrote at least one group of SCD patient was considered to have good perception of SCD. Each mention of SCD patient group was counted as one.

Since SCD has not been considered as a dental specialty in Indonesia, we hypothesized that specialist dentists were more "familiar" with SCD since dental specialist training would include dental management of complex cases like SCD patient. A correlation statistical analysis using Chi-Square test showed that specialist dentists demonstrated good SCD perception than general practitioners (Table 1). Other respondents' profiles such as gender, how long they have been practicing as dentists, and the year of their graduation did not show any correlation with SCD perception.

Table 1. Distribution of Dentist Competency and SCD Perception.

SND Perception	Dentistry Competency (N, %)		Total	*p*
	General Practioner	Specialist	N (%)	
**Poor**	110 (63.6%)	36 (46.8%)	146 (58.4)	0.019*
**Good**	63 (36.4%)	41 (53.2%)	104 (41.6)	

Chi-Square test, *$p < 0.05$

The majority of participants (72%) admitted of not performing SCD in their clinical practices. The most common reason provided by respondents (68.9%) was lack of experience in treating SCPs. Some respondents (31.1%) reporting inadequate surgical facilities (number of surgery or type of facilities) as the reason of difficulty to provide access or treatment to this

cohort. Others barriers to providing care included inadequate staff to cater for the special needs of such patients (23.9%), difficulty in managing behavioral problems associated with special care patients (21.7%), more time consuming (13.3%), no interest in treating special care patients or accessibility of the dental surgery (e.g. first floor practice) (9.4%), more staff time is needed (8.3%), high incidence of cancellation or broken appointments (4.4%), and others stated that they had never have SCPs coming to their dental surgery (Table 2).

Table 2. Distribution of Reason for not Practicing SCD.

Answer	Frequency (N=180)	%
Lack of experience in treating special care patients	124	68.9
No interest in treating special care patients	17	9.4
Inadequate staff to cater for the special needs of such patients	43	23.9
More staff time is needed	15	8.3
Treating special care patients is more time consuming	24	13.3
Too difficult to manage behavioral problems associated with special care patients	39	21.7
High incidence of cancellation or broken appointments	8	4.4
Inadequate surgical facilities (number of surgery or type of facilities)	56	31.1
Accessibility of the dental surgery (e.g. first floor practice)	17	9.4
Other	19	10.6

Respondents were given an opportunity to write their opinions in regards to SCD. Several translated responses in the free-text included the following:

- “There should be more introduction to SCD and its organization.”
- “I didn’t know about SCD before I participated in this survey.”
- “There should be more training or continuing education programs on SCD
- “Special care dentistry is important so dentist can provide care for patients who need SCD.”
- “Special care dentistry should be considered as a specialty or multidisciplinary collaboration.”
- “I thought that SCD was part of pediatric dentistry.”

Dentist' perception towards providing treatment to different categories of SCD Patients

Participants were asked to rate their perceived level of feeling and comfort in treating different groups of SCD patients. A majority of the participants felt positively in providing treatment to almost every groups of SCD patients except patients with infectious disease followed by patients with behavioral problem (Table 3). Those who treated elderly patients reported feeling "positive" (N=153, 61.2%). In terms of those who treated physically disabled patients, most rated their experiences either "positive" (N=159, 63.6%) or "very positive" (N=44, 17.6 %). When asked about their feelings in treating patient with intellectual disability and medically compromised patients, the majority of participants rated their experiences to be "positive" (N=133, 53.2% and N=121, 48.4%, respectively) or "very positive" (N=36, 14.4% and N= 31, 12.4%, respectively). In contrast, treating patients with infectious disease or treating patients with psychiatric or behavioral issues resulted in participants rating as either "negative" (N=99, 39.6% and N=86, 34,4%, respectively) or "positive" (N=73, 29.2% and N=92, 36,8%, respectively)".

Table 3. Distribution of Respondents' Perception of Feel towards Special Care Patients (N, %).

Special Care Patients	Extremely negative	Very negative	Negative	Positive	Very positive	Extremely positive
**Elderly**	1 (0.4)	0 (0)	29 (22.6)	153 (61.2)	46 (18.4)	21 (8.4)
**Physical disability**	1 (0.4)	2 (0.8)	28 (11.2)	159 (63.6)	44 (17.6)	16 (6.4)
**Intellectual disability**	3 (1.2)	12 (4.8)	54 (21.6)	133 (53.2)	36 (14.4)	12 (4.8)
**Medically-complex problem**	6 (2.4)	11 (4.4)	66 (26.4)	121 (48.4)	31 (12.4)	15 (6)
**Infectious disease**	21 (8.4)	31 (12.4)	99 (39.6)	73 (29.2)	17 (6.8)	9 (3.6)

**Behavior or psychological problem**	11 (4,4)	24 (9,6)	86 (34,4)	92 (36,8)	28 (11,2)	9 (3,6)

In terms of comfort (Table 4), most participants felt "comfortable" in treating elderly patients (N=176, 70.4%), patients with physical disability (N=169, 67.6%), intellectual disability (N=135, 54%), and patients with complex medical problems (N=121, 48.4%). However, most participants reported "uncomfortable" towards patients with infectious disease (N=119, 47.6%) and patients with behavior or psychological problem (N=114, 45.6%).

Table 4. Distribution of Respondents' Perception of Comfort towards Special Care Patients (N, %).

Special Care Patients	Extremely uncomfortable	Very uncomfortable	Uncomfortable	Comfortable	Very comfortable	Extremely comfortable
**Elderly**	1 (0.4)	1 (0.4)	34 (13.6)	176 (70.4)	29 (11.6)	9 (3.6)
**Physical disability**	1 (0.4)	3 (1.2)	43 (17.2)	169 (67.6)	25 (10)	9 (3.6)
**Intellectual disability**	3 (1.2)	11 (4.4)	86 (34.4)	135 (54)	11 (4.4)	4 (1.6)
**Medically-complex problem**	9 (3.6)	14 (5.6)	91 (36.4)	121 (48.4)	14 (5.6)	1 (0.4)
**Infectious disease**	38 (15.2)	42 (16.8)	119 (47.6)	44 (17.6)	7 (2.8)	0 (0)
**Behavior or psychological problem**	18 (7.2)	30 (12)	114 (45.6)	76 (30.4)	11 (4.4)	1 (0.4)

Government programs and initiatives for special care dentistry

Only a small number (N=5, 2%) of respondents answered that they knew about government program and initiatives for SCD and mentioned programs such as priority health services for elderly and people with disability in public health center or government's hospital

and financial support through the national health insurance. Of those who knew of such programs, they reported that they thought the programs were advantageous for SCPs.

Training programs for special care dentistry

Almost all participant dentists (N=243, 97.2%) felt that undergraduate dental students should receive didactic and clinical training in SCD and it should be given in their third year (42%) and fourth year (35.8%). Most respondents showed their willingness in attending continuing education programs in the topic of SCD (89.6%) and would consider attending postgraduate training in SCD (79.6%).

Referring special care patients to special care dentistry specialist

Table 5 showed the distribution of respondents' criteria in referring patient to SCD specialist. Their responses were almost similarly distributed for every criterion. However, 37 (14.8%) respondents chose that they would always refer SCP to a specialist when they need second opinion, followed by when they were treating patients with complex medical problems or medically compromised patients (N=35, 14%).

Table 5. Distribution of Special Care Patient Referral Criteria.

Criteria	Frequency (N,%)					
	Always	Very often	Often	Sometimes	Almost never	Never
**I would like a second opinion**	37 (14.8)	26 (10.4)	71 (28.4)	87 (34.8)	20 (8)	9 (3.6)
**I am uncomfortable performing the necessary procedure**	22 (8.8)	21 (8.4)	78 (31.2)	89 (35.6)	27 (10.8)	13 (5.2)
**I am unsure how to proceed with treatment for patient who is medically complex or compromised**	35 (14)	34 (13.6)	76 (30.4)	81 (32.4)	17 (6.8)	7 (2.8)
**Patient is intellectually disabled and unable to co-operate**	28 (11.2)	28 (11.2)	74 (29.6)	76 (30.4)	29 (11.6)	15 (6)
**Patient is physically disabled and I have no facilities**	29 (11.6)	24 (9.6)	75 (30)	78 (31.2)	28 (11.2)	16 (6.4)

**Patient has psychological problem which precludes treatment in a general dental surgery**	27 (10.8)	26 (10.4)	71 (28.4)	80 (32)	27 (10.8)	19 (7.6)
**Patient has behavioural problem which makes treatment delivery difficult**	30 (12)	31 (12.4)	76 (30.4)	73 (29.2)	26 (10.4)	14 (5.6)

Discussion

Many studies reported that oral disease and unmet dental needs were more prevalent among SCPs.[2–7] A significant number of barriers are faced by those patients with special needs. Amongst the most reported barriers in obtaining appropriate level of oral health care are economic burden, physical barriers, as well as social, behavioral, and educational factors.[29–33] Their disability and complicated health care needs may often prevent them from getting adequate dental care.[34–36] Limitation or lack of sensory or motoric coordination, attention to oral health, and dependence on others for their oral hygiene maintenance contributed to SCPs oral problems.[5,8,9,37–39] Achievement of non-compromised oral health in people with special needs requires removal of barriers that may limit the individual's capacity to undertake oral hygiene activities, exercise habits that promote oral health, and utilize oral health care services on a regular basis.[40] The lack of availability of dental practitioners is also a perceived barrier in accessing oral health care service.[41,42]

This study was conducted online to investigate the perception of dentists in Jakarta towards SCD using cross-adapted questionnaire. The data was collected in seven days using instant messaging application Whatsapp which ubiquitously used in Indonesia.

The use of Whatsapp as a method of recruitment for online epidemiological survey has been reported before.[43] Recruiting respondents by email is more common than Whatsapp in online survey, however both method were reported to have similar recruitment rate which were 28.1% and 24.9% respectively.[43] The response rate in this study was 20.3%, being quite similar with the previous published study which was conducted by email.[43]

More than half of the respondents in this study reported that they did not received SCD component during undergraduate program. Moreover, medically compromised patient was only recognized as part of SCD by 21 respondents. The undergraduate dentistry curriculum, however, according to the Indonesian Dentist Standard must include component of dental management of patients with complex medical health and patients with mental health issues.[44] This curriculum has been applied nationwide since 2011. Thus, this study showed that respondents were not aware that management of dental patients with complex medical condition as a part of SCD.

General dental practitioners have been trained in some tertiary dental curricula to provide care for special needs patients whose spectrum of need is less complicated.[15,45] However, they often find treating patients with special needs challenging[46] and often report difficulties with communication, thus creating sometimes seemingly insurmountable barriers to access of mainstream dentistry by this population.

It has been estimated that 90% of people requiring SCD can receive treatment in local, primary care centers[47]; however, this was dependent upon adequate education and training of the dental team.[26] Curricula design is the key in achieving the required level of knowledge and skills during undergraduate education.[48] Subjects like Special Needs/Care Dentistry would be the key elements in undergraduate training and ought to be given more emphasize, especially with the increased life expectancy amongst this population.[49]

The findings that most respondents in this study cannot described SCD was not completely unexpected since the term SCD itself was relatively new in Indonesia. This result was in parallel with the finding among Malaysian dental students who were unable to define SCD.[24] However, SCD has starting to gain recognition as a specialty in Malaysia.[21,24] Undergraduate dental curriculum in Malaysia also had included SCD as an integrated module and students were exposed to SCPs through rotation in SCD clinics and visitations to

institutions caring for SCPs.[26] These efforts could also be applied in Indonesia so Indonesian dental students can gain understanding of SCD and later become competent oral care provider to SCPs.

To the best of our knowledge, whether SCD should be a specialty or a special interest group consisted of dentists with different specialty background was still under discussion in Indonesia. The matter of discussion was whether SCD can show the specific knowledge and set of skills which has not existed in any other specialty in dentistry. An editorial article in 2016 argued that SCDA's definition of SCD can be fulfilled by the specialties that have been recognized by the American Dental Association (ADA), as well as by many non-specialty in dentistry.[17] But, no matter with the status of SCD in Indonesia, it did not changed the fact that oral health care needs of SCPs population was being neglected.[6,7] This is a larger issue that should be addressed.

Most of the study respondents said that they did not perform SCD in their daily practice. Many cited their lack of experience in treating SCPs as the reason, although they generally felt positive and comfortable toward treating SCPs. These results were similar with the results reported in Australia as well as in other studies.[13,36,40] This study thus provided another evidence that there is an increased need of SCD development among dental professionals.

A recent study about the attitude of dental students toward treating SCPs in Austria showed that implementation of SCD in dental school positively affect students' perception towards SCPs although it did not positively affect their confidence to treat these patients.[50] This should come as a notice to the dental community, especially educators, that SCD component in dental school alone is not necessarily sufficient to prepare students as dentists who can or willing to practice SCD.

It is well reported that dental caries and periodontal disease have a strong correlation with poor oral hygiene. It is not surprising that individuals with special needs were also

consistently shown to have more oral disease.[35,45,51–57] In addition, poor oral health including greater restorative procedures and minimal preventive treatment were common amongst those with intellectually disability in the presence or absence of physical disability.[36] Several studies reported that individuals with intellectual disability were more likely to receive more tooth extractions rather than restorations for dental problems, compared with individuals in the general population.[58,59] Since SCPs highly dependent on their caregivers' perception toward oral health, this could be the reason of the lack of SCPs in dental practice encountered by the participants in this study. It is documented that caregivers of SCP understood that oral care was important, however, there were still some who thought that it was not essential and dental visits should only be performed when the patients were in pain.[60] Preventive measure for this cohort will always require the cooperation, positive attitude, and continued interest and treatment priorities of the caregivers. In addition, caregivers' knowledge of oral hygiene measures impacted on the degree of appropriate oral care provided to their clients. Unfortunately, many caregivers received minimal training in provision of oral care. Lack of supervision and negative attitudes toward dental health by the caregiver have been cited as the obstacles to good oral health.[35]

The WHO has published a World Disability Report stated that around 15% or over a billion people in the world have a kind of disability.[61] With the increasing life expectancy of SCPs, this number was expected to increase.[10] Therefore, the demand of SCD will also grow in the future and dentists should expect and be prepared to deliver SCD in their dental practice.

Particularly with young SCPs, pediatric dentistry specialists can provide oral health care to this population. However, these patients will reach the age of adulthood and no longer be in the care of pediatric dentists so they could encounter unmet oral care needs.[10,21] In areas where there were a lack of SCD dentists, SCPs may receive oral and dental care from the

general practitioners.[10,13,24] This was no different in the present study especially considering that this specialty is not recognized yet in Indonesia.

The general dentists would rely on their undergrad training or continuous professional development courses attended post-graduation to manage this patient cohort. However, there has been report that not all dentists were willing or able to treat SCPs and this has become the greatest barrier in getting oral healthcare in SCPs.[10] It has also been reported that many respondents in this study would like a second opinion when they treat SCPs. However, there is still no or very minimum treatment pathway for them to follow so far.

Despite the poor perception, most respondents in this study showed interest and motivation in SCD. They expressed their willingness to attend continuing education in SCD. Previous studies showed that dentists will feel more confident and ready to treat SCPs when they had adequate training in their undergraduate training and through continuing education.[62,63] This is also in accordant to the study of dental students in Austria, that adequate training should also be given to develop confidence.[50] One study also reported that dentists who had positive perception towards their dental education (i.e. satisfied with SCD education), they were more likely to show positive perception in providing oral health care to SCP.[62] With more dentists willing and prepared to treat SCPs, the gap of unmet oral health care can be reduced therefore improving patients' quality of life.[64]

Lastly, this study was a self-recruitment study so the result should be cautiously interpreted and not to be generalized. The respondents who recruited themselves into this study were usually those who have interest towards the topic, i.e. SCD, thus they might have given biased responses in the survey. But, this study was an "exploratory" study on a niche topic, therefore a survey that was distributed directly to target population which they could subsequently volunteer to become respondents was a simple, fast, and a cost-efficient method.

Conclusion

This study explored the perception of SCD amongst the dentists in Jakarta using survey conducted online. Most respondents had poor perception in SCD, reported lack of education in SCD, and did not deliver SCD in their dental practice. This showed that more efforts should be initiated to improve the dental education and to raise the awareness of SCD in Indonesia, not only for dentists but also for the community of SCPs. For starters, it can begin by a collaboration between the Indonesian Dental Council, dental schools, and ISSCD to promote training and education in the area of SCD. Furthermore, this study can be followed up with investigation in the oral health care need of various SCPs to convey the importance of SCD and uncover the barriers to receiving dental care.

Acknowledgment

Authors would like to thank Dr. Melissa Adiatman for statistical consultation.

Funding

The study and publication of this article received support from Universitas Indonesia through International-Indexed Publication Grant (PUTI Universitas Indonesia).

Conflict of interest

None declared.

References

1. Rahardjo A, Maharani DA. A review of Indonesia's dental health - past, present and future. Int J Clin Prev Dent. 2014;10(3):121-126.

2. Gil-Montoya JA, Sánchez-Lara I, Carnero-Pardo C, Fornieles-Rubio F, Montes J, Barrios R, et al. Oral hygiene in the elderly with different degrees of cognitive impairment and dementia. J Am Geriatr Soc. 2017;65(3):642-647.

3. Pini D de M, Fröhlich PCGR, Rigo L. Oral health evaluation in special needs individuals. Einstein (Sao Paulo). 2016;14(4):501-507.

4. Lee J-Y, Lim K-C, Kim S-Y, Paik H-R, Kim Y-J, Jin B-H. Oral health status of the disabled compared with that of the non-disabled in Korea: A propensity score matching analysis. PLoS One. 2019;14(1):e0208246.

5. Kalyoncu IÖ, Tanboga I. Oral health status of children with autistic spectrum disorder compared with non-authentic peers. Iran J Public Health. 2017;46(11):1591-1593.

6. Trihandini I, Wiradidjaja Adiwoso A, Erri Astoeti T, Marks L. Oral health condition and treatment needs among young athletes with intellectual disabilities in Indonesia. Int J Paediatr Dent. 2012;23(6):n/a-n/a.

7. Ningrum V, Wang WC, Liao HE, Bakar A, Shih YH. A special needs dentistry study of institutionalized individuals with intellectual disability in West Sumatra Indonesia. Sci Rep. 2020;10(1).

8. Kebede B, Kemal T, Abera S. Oral health status of patients with mental disorders in Southwest Ethiopia. PLoS One. 2012;7(6):e39142.

9. Naidoo M, Singh S. The oral health status of children with autism spectrum disorder in

KwaZulu-Nata, South Africa. BMC Oral Health. 2018;18(1):165.

10. Williams JJ, Spangler CC, Yusaf NK. Barriers to dental care access for patients with special needs in an affluent metropolitan community. Spec Care Dent. 2015;35(4):190-196.

11. Kementerian Kesehatan Republik Indonesia. *Situasi Penyandang Disabilitas*. Jakarta: Pusat Data dan Informasi Kementerian Kesehatan Republik Indonesia; 2014.

12. World Bank. Indonesia. https://data.worldbank.org/country/ID. Accessed August 9, 2020.

13. Derbi HA, Borromeo GL. The perception of special needs dentistry amongst general dentists within Western Australia, Australia. J Gerontol Geriatr Res. 2016;5(4):1-8.

14. American Academy of Pediatric Dentistry. Definition of special health care needs. Pediatr Dent. 2016;38(special issue):16.

15. Ettinger RL, Chalmers J, Frenkel H. Dentistry for persons with special needs: how should it be recognized? J Dent Educ. 2004;68(8):803-806.

16. Ferrand F. Defining the population requiring special care dentistry using the International Classification of Functioning, Disability and Health. J Disabil Oral Heal. 2006;7:143.

17. Ackerman MB. Specialist envy: Is special care dentistry a dental specialty or a dental special interest group? Spec Care Dent. 2016;36(1):3-6.

18. Queen AN. Evidence-based dentistry and its role in caring for special needs patients. Dent Clin North Am. 2016;60(3):605-611.

19. Mugayar L, Hebling E, Dias PV. Special care dentistry: A new specialty in Brazil.

Spec Care Dent. 2007;27(6):232-235.

20. Scully C, Ettinger RL. The influence of systemic diseases on oral health care in older adults. J Am Dent Assoc. 2014;138:S7-S14.

21. Hamzah S. Special needs dentistry (Snd) in Malaysia: a way forward. Malays Dent J. 2012;34(1):33-36.

22. Borromeo GL, Ahmad MS, Buckley S, Bozanic M, Cao A, Al-Dabbagh M, et al. Perception of Special Needs Dentistry education and practice amongst Australian dental auxiliary students. Eur J Dent Educ. 2018;22(3):e321-e326.

23. Bindal P, Lin CW, Bindal U, Safi SZ, Zainuddin Z, Lionel A. Dental treatment and special needs patients (SNPs): Dentist's point of view in selected cities of Malaysia. Biomed Res. 2015;26(1):152-156.

24. Ahmad MS, Razak IA, Borromeo GL. Special needs dentistry: Perception, attitudes and educational experience of Malaysian dental students. Eur J Dent Educ. 2015;19(1):44-52.

25. Abed H. Special care dentistry in Saudi Arabia: Development as a dental specialty – an opinion paper. J Disabil Oral Heal. 2017;18(January):61-67.

26. Fuad NA, Jacob J, Koh WT, Mani SA, Lim W-LS, Wong CS, et al. Special care dentistry curriculum at the undergraduate level: Students' perspective. J Dent Indones. 2015;22(3):75-79.

27. PB PDGI. http://pdgi.or.id/halaman/ikatan-peminatan. Accessed August 9, 2020.

28. Beaton DE, Bombardier C, Guillemin F, Ferraz MB. Guidelines for the process of cross-cultural adaptation of self-report measures. Spine (Phila Pa 1976). 2000;25(24):3186-3191.

29. Rapalo DM, Davis JL, Burtner P, Bouldin ED. Cost as a barrier to dental care among people with disabilities: a report from the Florida behavioral risk factor surveillance system. Spec Care Dent. 2010;30:133-139.

30. Rouleau T, Harrington A, Brennan M, Hammond F, Hirsch M, Nussbaum M, et al. Receipt of dental care and barriers encountered by persons with disabilities. Spec Care Dent. 2011;31(2):63-67.

31. Yuen HK, Wolf BJ, Bandyopadhyay D, Magruder KM, Selassie AW, Salinas CF. Factors that limit access to dental care for adults with spinal cord injury. Spec Care Dent. 2010;30(4):151-156.

32. Dougall A, Fiske J. Access to special care dentistry, part 1. Access. Br Dent J. 2008;204(11):605-616.

33. Siegal MD. Dentists' reported willingness to treat disabled patients. Spec Care Dent. 1985;5(3):102-108.

34. Waldman HB, Perlman SP. Disability and rehabilitation: Do we ever think about needed dental care? A case study: The USA. Disabil Rehabil. 2010;32(11):947-951.

35. Cumella S, Ransford N, Lyons J, Burnham H. Needs for oral care among people with intellectual disability not in contact with Community Dental Services. J Intellect Disabil Res. 2000;44(1):45-52.

36. Pezzementi ML, Fisher MA. Oral health status of people with intellectual disabilities in the southeastern United States. J Am Dent Assoc. 2005;136(7):903-912.

37. Bertaud-Gounot V, Kovess-Masfety V, Perrus C, Trohel G, Richard F. Oral health status and treatment needs among psychiatric inpatients in Rennes, France: a cross-sectional study. BMC Psychiatry. 2013;13(1):227.

38. Levi LE, Lalla R V. Dental Treatment Planning for the Patient with Oral Cancer. Dent Clin North Am. 2018;62(1):121-130.

39. Mansoor D, Al Halabi M, Khamis AH, Kowash M. Oral health challenges facing Dubai children with autism spectrum disorder at home and in accessing oral health care. Eur J Paediatr Dent. 2018;19(2):127-133.

40. Pradhan A, Slade GD, Spencer AJ. Factors influencing caries experience among adults with physical and intellectual disabilities. Community Dent Oral Epidemiol. 2009;37(2):143-154.

41. Pradhan A, Slade G, Spencer A. Access to dental care among adults with physical and intellectual disabilities: Residence factors. Aust Dent J. 2009;54(3):204-211.

42. Smith BJ, Ghezzi EM, Manz MC, Markova CP. Perceptions of oral health adequacy and access in Michigan nursing facilities. Gerodontology. 2008;25(2):89-98.

43. Blumenberg C, Menezes AMB, Gonçalves H, Assunção MCF, Wehrmeister FC, Barros AJD. How different online recruitment methods impact on recruitment rates for the web-based coortesnaweb project: A randomised trial. BMC Med Res Methodol. 2019;19(1):127.

44. Konsil Kedokteran Indonesia. *Standar Kompetensi Dokter Gigi Indonesia*. Jakarta: Konsil Kedokteran Indonesia; 2015.

45. Gallagher JE, Fiske J. Special Care Dentistry: A professional challenge. Br Dent J. 2007;202(10):619-629.

46. Ferguson FS, Berentsen B, Richardson PS. Dentists' willingness to provide care for patients with developmental disabilities. Spec Care Dent. 1991;11(6):234-237.

47. Joint Advisory Committee for Special Care Dentistry. *A Case of Need: Proposal for a*

Specialty in Special Care Dentistry. London: JACSCD; 2003.

48. Bullock A, Frame J, Holmlund A, Limanowska-Shaw H, Shaw G. Are Polish and Swedish dental graduates adequately prepared for dental practice in the UK? A discussion of the transferability of general dental practitioners in Europe. Eur J Dent Educ. 2002;6(2):49-53.

49. Atkin PA, Cunningham A, Andrews L. Special care dentistry trainee views on the medical and oral medicine elements of the specialist training curriculum. Eur J Dent Educ. 2020;24(1):36-41.

50. Holzinger A, Lettner S, Franz A. Attitudes of dental students towards patients with special healthcare needs: Can they be improved? Eur J Dent Educ. 2020;24(2):243-251.

51. Traci MA, Seekins T, Szalda-Petree A, Ravesloot C. Assessing secondary conditions among adults with developmental disabilities: A preliminary study. Ment Retard. 2002;40(2):119-131.

52. Owens PL, Kerker BD, Zigler E, Horwitz SM. Vision and oral health needs of individuals with intellectual disability. Ment Retard Dev Disabil Res Rev. 2006;12(1):28-40.

53. Shyama M, Al-Mutawa SA, Honkala S, Honkala E. Supervised toothbrushing and oral health education program in Kuwait for children and young adults with Down syndrome. Spec Care Dent. 2003;23(3):94-99.

54. Australian Institute of Health and Welfare. *Chronic Conditions and Oral Health. Research Report Series No. 56. Cat. No. DEN 221.* Canberra; 2012.

55. López-Pérez R, Borges-Yáñez SA, Jiménez-García G, Maupomé G. Oral hygiene,

gingivitis, and periodontitis in persons with Down syndrome. Spec Care Dent. 2002;22(6):214-220.

56. Hennequin M, Allison PJ, Veyrune JL. Prevalence of oral health problems in a group of individuals with Down syndrome in France. Dev Med Child Neurol. 2000;42(10):691-698.

57. Feldman CA, Giniger M, Sanders M, Saporito R, Zohn HK, Perlman SP. Special olympics, special smiles: Assessing the feasibility of epidemiologic data collection. J Am Dent Assoc. 1997;128(12):1687-1696.

58. Pregliasco F, Ottolina P, Mensi C, Carmagnola D, Giussani F, Abati S, et al. Oral health profile in an institutionalized population of Italian adults with mental retardation. Spec Care Dent. 2001;21(6):227-231.

59. Vázquez CR, Garciflan MR, Rioboo R, Bratos E. Prevalence of dental caries in an adult population with mental disabilities in Spain. Spec Care Dent. 2002;22(2):65-69.

60. Shah AH, Naseem M, Khan MS, Asiri FYI, AlQarni I, Gulzar S, et al. Oral health knowledge and attitude among caregivers of special needs patients at a Comprehensive Rehabilitation Centre: an analytical study. Ann Stomatol (Roma). 2017;8(3):110.

61. World Health Organization. Disability. https://www.who.int/health-topics/disability. Accessed July 9, 2020.

62. Alamoudi NM, Almushayt AS, El Derwi DA, Mirdad LH, El Ashiry EA. Dentists' perception of the care of patients with special health care needs in Jeddah, Saudi Arabia. J Oral Hyg Heal. 2017;5(3):1-7.

63. Dao LP, Zwetchkenbaum S, Inglehart MR. General dentists and special needs patients: Does dental education matter? J Dent Educ. 2005;69(10):1107-1115.

64. Faulks D, Freedman L, Thompson S, Sagheri D, Dougall A. The value of education in special care dentistry as a means of reducing inequalities in oral health. Eur J Dent Educ. 2012;16(4):195-201.

65. Khazaal Y, Van Singer M, Chatton A, Achab S, Zullino D, Rothen S, et al. Does self-selection affect samples'representativeness in online surveys an investigation in online video game research. J Med Internet Res. 2014;16(7).

Figure 1

Click here to access/download;Figure;Fig1.tiff

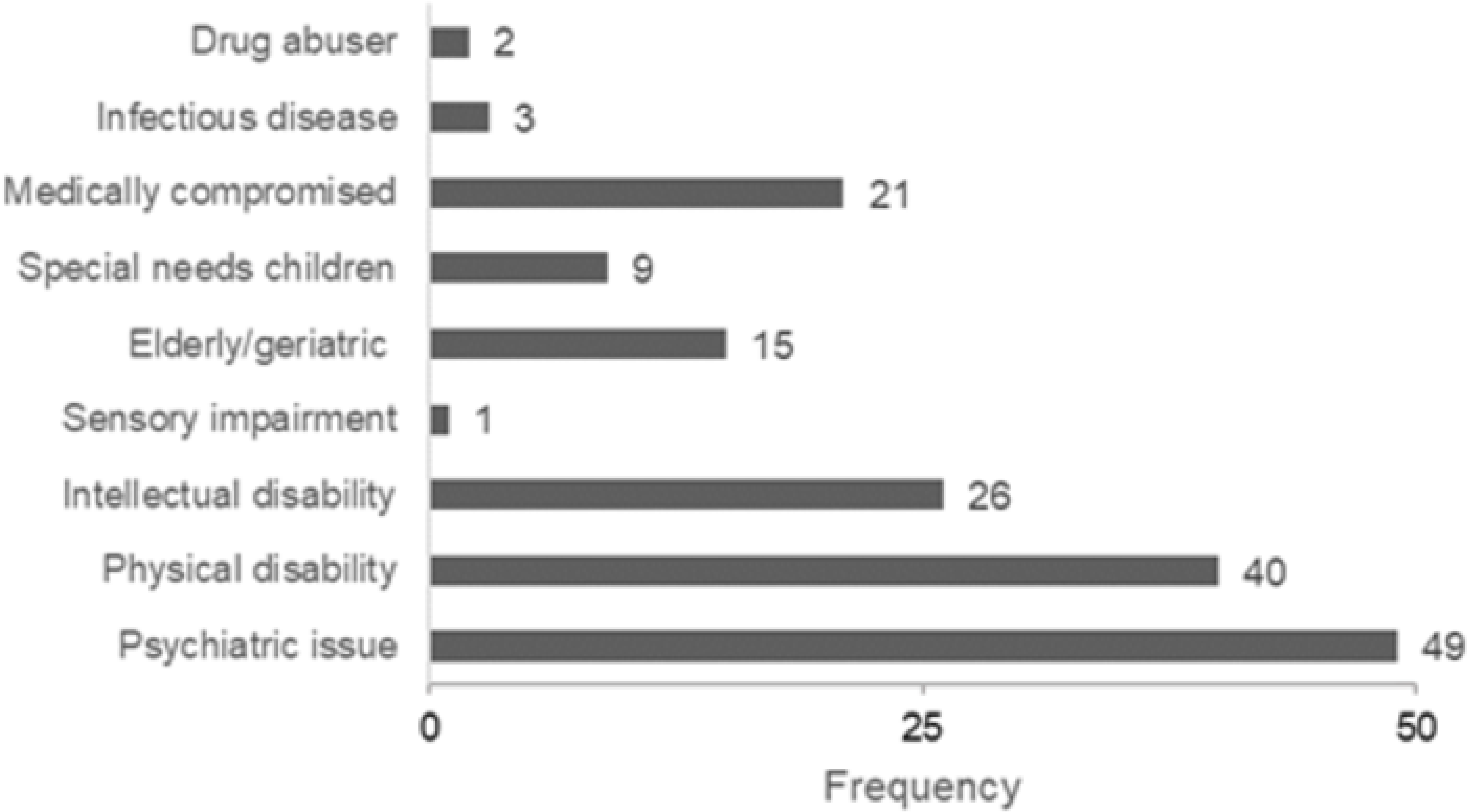

Supplement: Attachment — Submitted filename: PONE-D-20-32506_reviewer.pdf [file pone.0249727.s001.pdf]
