# Supplementary material for: Special care dentistry perception among dentists in Jakarta: An online survey study
Source: PLoS One. 2021 Apr 7;16(4):e0249727. doi: 10.1371/journal.pone.0249727 (PMC8026065; doi:10.1371/journal.pone.0249727)
Supplement: Attachment — Submitted filename: RESPONSE TO REVIEWERS.docx [file pone.0249727.s002.docx]

**RESPONSE TO REVIEWERS**

Dear Academic Editor,

The authors would like to thank the reviewers for their comments which help to improve the manuscript. In the following, we addressed all the comments, questions, and suggestions by the reviewers to the manuscript submitted by Mandasari et al, “Special care dentistry perception among dentists in Jakarta: An online survey study” (Manuscript ID: PONE-D-20-32506). We believed that this manuscript has been improved. As of editor’s instruction, we provide manuscript file which highlighted all the changes using track changes feature. Comments from reviewers are typed in italics and colored black, while authors’ response is typed in blue.

Contents

[**Editor**	1](#_Toc65887153)

[**Reviewer #1**	3](#_Toc65887154)

[**Reviewer #2**	3](#_Toc65887155)

# **Editor**

*Please ensure that your manuscript meets PLOS ONE's style requirements, including those for file naming.*

We apologize for this error. We have carefully read the style requirements of Plos One (https://journals.plos.org/plosone/s/revising-your-manuscript) and revised the manuscript as well as the naming of the files.

Response to reviewers is named “Response to reviewers.docx”

Revised manuscript (marked up copy) is named as “Revised Article with Changes Highlighted.docx”

Revised manuscript (clean copy) is named “Manuscript.docx”

Revised title page file is named “Title page.docx”

Revised cover letter is named “Cover letter.docx”

Figure 1 which has been submitted to PACE and met all the requirements is named “Fig1.TIF”

*Please provide additional details regarding participant consent. In the ethics statement in the Methods and online submission information, please ensure that you have specified (1) whether consent was informed and (2) what type you obtained (for instance, written or verbal, and if verbal, how it was documented and witnessed). If your study included minors, state whether you obtained consent from parents or guardians. If the need for consent was waived by the ethics committee, please include this information.*

We added sentences regarding participation informed consent as suggested by the editor. The added sentences are as follows:

“The online questionnaire included informed consent page and only respondents who agreed to participate would continue to complete the questionnaire. The responses were recorded electronically as text.”

To elaborate, the questionnaire was made as a Google Form with multiple pages (subsections). The first page was information regarding the survey including statement of respondents’ inclusion and exclusion criteria, objective of the study, respondents’ confidentiality, as well as main researcher contact info. The respondents who chose to participate in the study opt for the answer “yes” when being asked whether they would like to enroll in the study. After this participation confirmation is obtained, the respondents can proceed to the subsequent pages and complete the survey.

*If you are reporting a retrospective study of medical records or archived samples, please ensure that you have discussed whether all data were fully anonymized before you accessed them and/or whether the IRB or ethics committee waived the requirement for informed consent. If patients provided informed written consent to have data from their medical records used in research, please include this information.*

This study does not include any medical records. In the raw data set that we upload into the repository, we have removed any variables which may identify the respondents’. However, the removal of this variable from the data set does not alter the reproducibility of the data set.

*We note that you have indicated that data from this study are available upon request. PLOS only allows data to be available upon request if there are legal or ethical restrictions on sharing data publicly. For information on unacceptable data access restrictions, please see* [*http://journals.plos.org/plosone/s/data-availability#loc-unacceptable-data-access-restrictions*](http://journals.plos.org/plosone/s/data-availability#loc-unacceptable-data-access-restrictions)*.*

We have deposited the data set used for analysis in this manuscript as well as additional files such as the questionnaire used in this study in the suggested repository. Currently the data is limited for review purposes and can be access at:

https://datadryad.org/stash/share/LaW1ffJpEc4W96joEutqVnJPT2rst0uxhAFk3WuzV0g.

*In your revised cover letter, please address the following prompts:*

*a) If there are ethical or legal restrictions on sharing a de-identified data set, please explain them in detail (e.g., data contain potentially identifying or sensitive patient information) and who has imposed them (e.g., an ethics committee). Please also provide contact information for a data access committee, ethics committee, or other institutional body to which data requests may be sent.*

*b) If there are no restrictions, please upload the minimal anonymized data set necessary to replicate your study findings as either Supporting Information files or to a stable, public repository and provide us with the relevant URLs, DOIs, or accession numbers. Please see http://www.bmj.com/content/340/bmj.c181.long for guidelines on how to de-identify and prepare clinical data for publication. For a list of acceptable repositories, please see http://journals.plos.org/plosone/s/data-availability#loc-recommended-repositories.*

*We will update your Data Availability statement on your behalf to reflect the information you provide.*

We have revised the cover letter. The letter now included that the data set can be accessed during review period using this URL:

https://datadryad.org/stash/share/LaW1ffJpEc4W96joEutqVnJPT2rst0uxhAFk3WuzV0g

# **Reviewer #1**

*The manuscript entitled "Special Care Dentistry Perception among Dentists in Jakarta: An Online Survey Study" poses an interesting question highlighting a somewhat neglected, yet important, dentistry field. The limitation is the sample size.*

We greatly appreciate reviewer #1 for reviewing our manuscript. We are delighted and honored that reviewer #1 agrees that the theme of this manuscript has a merit. We also agree and realized that the sample size in this study is low as also pointed out by reviewer #2. This is indeed a limitation of this study.

Web based survey are increasing in popularity and have variable response rate. These variations are influenced by the modes of survey, i.e. emails/web/social media/etc/combined modes. In this study, the response rate was 20.3%. James et al (2011) mentioned that the community standard for physicians’ response rate was 50%. Weaver et al (2019) employed survey using combined method of either mail or web survey and combination of web-mail. Their overall response was 18.9%, highest response rate was obtained by mail only and web-mail (19%) while the web only method was 15%. Study by Blumenberg (2019) was also carried out using web, in particular through Whatsapp, similar to our study. Their response rate was 24.9% and we thought that this number is similar to our response rate. They also performed survey using recruitment through Facebook and emails which yielded higher response rate (30.6% and 28.1% respectively). However, these methods were not feasible in our study. We contacted the Indonesian Dental Association and we were unable to obtained the data of emails and recruiting through Facebook would be difficult to determine the response rate since we have no means to calculate the number of the viewers. We have yet found other survey using Whatsapp to directly compare response rates.

However, despite the limitation caused by low response rate, we thought that the respondents included in this study showed sufficient representation since we distributed the questionnaire throughout dentist groups in all five cities in the province of Jakarta, we also have good balance between general practioner dentists and specialist dentists, and a wide range of respondents age. We hope that these representations can somewhat reduce the nonresponse bias in this study. Moreover, as many as 250 respondents participated in this study. We conducted an analysis using G Power software to calculate the minimal number of samples in this study and obtained a minimal number of 200. Even though the overall response rate was low, we have exceeded the minimal sample number for adequate analysis.

Screenshot of G Power software in calculating the minimal number of sample.

# **Reviewer #2**

*Please, find my comments in the attached file (PDF file of the manuscript). All my comments could be seen in the attached file (PDF file of the manuscript). The authors should fulfill all my comments in the manuscript.*

We sincerely thank reviewer #2 for reviewing our manuscript and providing instruction to make this manuscript better. We have addressed the comments found in the PDF files. Our response to the comments are listed below.

Abstract:

*The year of study and statistical methods should be mentioned.*

We have added the year of study and statistical method used in this study in the method subsection in the abstract of the revised manuscript.

Line 53:

*Disability data of other countries should be written.*

We added worldwide disability data as reported by WHO. We hope that this statement is sufficient to address your concern. The addition is preceding the data of disability in Indonesia:

“The WHO estimated that roughly 15% of world population has some kind of disability which translated to over one billion people and this number is projected to be increasing in the future, partially due to the ageing societies and increase in chronic health conditions.”

Line 102:

*The details of the questionnaire should be explained in the method.*

We apologize that the details of the questionnaire were not stated clearly in the manuscript. We have upload the questionnaire in data repository service recommended by Plos One and added the statement “The questionnaire used in this study can be accessed from the public repository” in the manuscript so interested readers can obtain the questionnaire used in this study.

The data repository can be accessed during review period using this link:

https://datadryad.org/stash/share/LaW1ffJpEc4W96joEutqVnJPT2rst0uxhAFk3WuzV0g

The data set will be made publicly available once the manuscript is accepted for publication (doi:10.5061/dryad.vhhmgqnsg)

Line 123:

*Response rate is very low.*

We realized and agree with the reviewer that the response rate in this study was low. This observation was also made by reviewer #1. Web based survey are increasing in popularity and have variable response rate. These variations are influenced by the modes of survey, i.e. emails/web/social media/etc/combined modes. In this study, the response rate was 20.3%. James et al (2011) mentioned that the community standard for physicians’ response rate was 50%. Weaver et al (2019) employed survey using combined method of either mail or web survey and combination of web-mail. Their overall response was 18.9%, highest response rate was obtained by mail only and web-mail (19%) while the web only method was 15%. Study by Blumenberg (2019) was also carried out using web, in particular through Whatsapp, similar to our study. Their response rate was 24.9% and we thought that this number is similar to our response rate. They also performed survey using recruitment through Facebook and emails which yielded higher response rate (30.6% and 28.1% respectively). However, these methods were not feasible in our study. We contacted the Indonesian Dental Association and we were unable to obtained the data of emails and recruiting through Facebook would be difficult to determine the response rate since we have no means to calculate the number of the viewers. We have yet found other survey using Whatsapp to directly compare response rates.

However, despite the limitation caused by low response rate, we thought that the respondents included in this study showed sufficient representation since we distributed the questionnaire throughout dentist groups in all five cities in the province of Jakarta, we also have good balance between general practioner dentists and specialist dentists, and a wide range of respondents age. We hope that these representations can somewhat reduce the nonresponse bias in this study. Moreover, as many as 250 respondents participated in this study. We conducted an analysis using G Power software to calculate the minimal number of samples in this study and obtained a minimal number of 200. Even though the overall response rate was low, we have exceeded the minimal sample number for adequate analysis.

Screenshot of G Power software in calculating the minimal number of sample.

Line 154-156:

*This sentence and other similar sentiences in the Result part should write in the Discussion Part of the manuscript. In the result part only results of the study without any interpretation should be written.*

We thank the reviewer for pointing this out and we apologize for this mistake. We have removed such sentences from the result subsection.

Table 4 (Line 210-211):

Typographical error marked with yellow highlights of the overflowing column title

We have decreased the font size. Thank you for pointing this error.

Line 248-250:

*Method sentence. It should be deleted.*

We thank the reviewer for pointing this mistake. We have deleted the sentence from the discussion subsection and incorporated the details to the method section.

Line 378:

*The number of references should be reduced.*

We agree with the reviewer. Our approach in this matter was by identifying and removing redundant references and/or older references which also included the removal some part of the manuscript without changing the main ideas. Previously, there were 65 references and it has been reduced into 44. We expect this changes improve the conciseness of the manuscript.

We would like to **thank all the referees again** for taking the time to review our manuscript. Despite the limitation, we believe that this manuscript discussed important findings which show merit especially in the development of special care dentistry in Indonesia. This field of dentistry is currently underserved while the demand will increase in the near future.

Thank you for your kind attention. We are looking forward for further comments.

Sincerely,

Masita Mandasari
